# Grief, Emotion and Infection

**Published:** 1895-02

**Authors:** 


### Grief, Emotion, and Infection.

That grief prostrates, often causing physical disease and sometimes death, has long been a matter of every-day knowledge. The way in which such effects are brought about has been the subject of careful study by an investigator named Bassi, who has recorded observation on animals which apparently died in consequence of capture. Birds, moles, and a dog, finally succumbed to conditions that correspond in the human animal to acute nostalgia and a "broken heart." These humble cousins of the human race were examined post-mortem.

Generally there was hyperemia, says *The Lancet*, sometimes associated with capillary hemorrhages of the abdominal organs, more especially of the liver, with fatty and granular degeneration of their elements, and sometimes bile was found in the stomach with or without a catarrhal condition. The clinical symptoms were at first those of excitement, especially in the birds, and followed by depression and persistent anorexia. The theory suggested by Dr. Bassi is that the nervous disturbance interferes with the proper nutrition of the tissues in such a way as to give rise to poisonous substances—ptomaines—which set up acute degeneration of the parenchymatous elements similar to that which occurs in consequence of the action of certain poisonous substances, as phosphorus, or to that met with in some infectious disease. In support of this view, it may be remembered that Shule earlier found parenchymatous degeneration in persons dead from acute delirium, and that Zenker found hemorrhages into the pancreas in persons who had died suddenly.—*N. Y. Medical Record.*

